# Interfacing antibody-based microarrays and digital holography enables label-free detection for loss of cell volume

**DOI:** 10.4155/fso.14.2

**Published:** 2015-11-01

**Authors:** Zahra El-Schich, Emmy Nilsson, Anna S Gerdtsson, Christer Wingren, Anette Gjörloff Wingren

**Affiliations:** 1 Department of Biomedical Science, Health & Society, Malmö University, Malmö, Sweden; 2 Department of Immunotechnology & CREATE Health, Medicon Village, Lund University, Lund, Sweden

**Keywords:** antibody, cellular, holography, microarray, volume

## Abstract

**Background:**

We introduce the combination of digital holographic microscopy (DHM) and antibody microarrays as a powerful tool to measure morphological changes in specifically antibody-captured cells. The aim of the study was to develop DHM for analysis of cell death of etoposide-treated suspension cells.

**Results/methodology:**

We demonstrate that the cell number, mean area, thickness and volume were noninvasively measured by using DHM. The cell number was stable over time, but the two cell lines showed changes of cell area and cell irregularity after treatment. The cell volume in etoposide-treated cells was decreased, whereas untreated cells showed stable volume.

**Conclusion:**

Our results provide proof of concept for using DHM combined with antibody-based microarray technology for detecting morphological changes in captured cells.

## Abstract (lay abstract)

We use an innovative technique that combines digital holographic microscopy and the capture of cells with antibody microarrays as a powerful tool to measure cellular morphological changes. With this technique, the cells can be noninvasively viewed in 3D over time. We obtain results showing changes in cellular parameters including cell area, thickness and volume of the captured cells after treatment with the cell death-inducing drug etoposide. The cell volume in etoposide-treated cells showed a decrease, while untreated cells remained stable. Digital holographic microscopy combined with antibody microarray technology can be a future method for detecting morphological changes in treated cancer cells.

**Figure 1.**
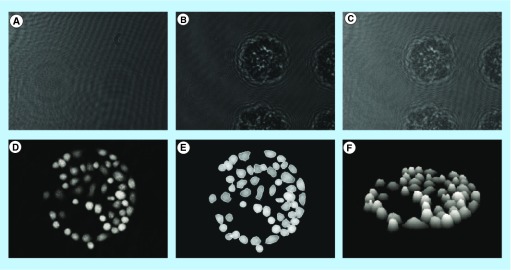
**Jurkat cells captured on antibody Lewis X Clone-1.** **(A)** Reference diffraction pattern; **(B)** object diffraction pattern and **(C)** hologram diffraction pattern; **(D)** numerical reconstruction of the hologram rendered the 3D image of the cells; **(E)** segmentation algorithm marked each individual cell; and **(F)** 3D image of the Jurkat cells.

**Figure 2.**
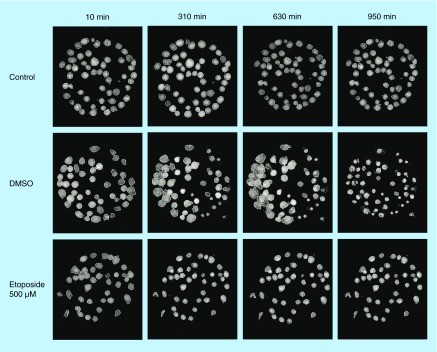
**Hologram images of Jurkat cells captured on antibody Lewis X Clone-1.** The images are showing control, DMSO and etoposide-treated cells at four different timepoints: 10, 310, 630 and 950 min. The cells are segmented to analyze cell parameters. DMSO: Dimethyl sulfoxide.

**Figure 3.**
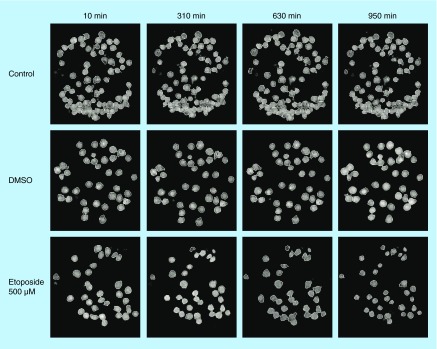
**Hologram images of U2932 cells captured on antibody HLA-DR.** The images are showing control, DMSO and etoposide-treated cells at four different timepoints: 10, 310, 630 and 950 min. The cells are segmented to analyze cell parameters. DMSO: Dimethyl sulfoxide

**Figure 4.**
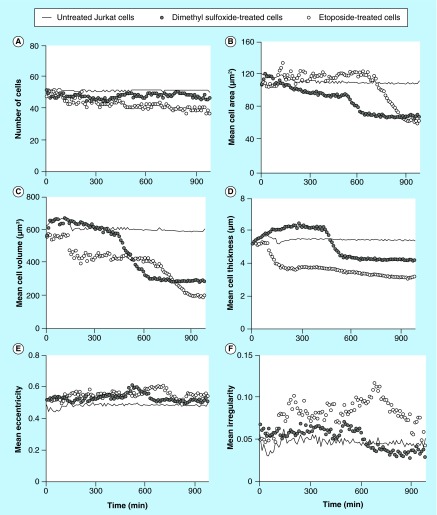
**Different cellular parameters of treated (etoposide or dimethyl sulfoxide) or untreated Jurkat cells captured on antibody Lewis X Clone-1 were analyzed over time.** **(A)** Number of cells; **(B)** mean cell area; **(C)** mean cell volume; **(D)** mean cell thickness; **(E)** mean cell eccentricity; and **(F)** mean cell irregularity.

**Figure 5.**
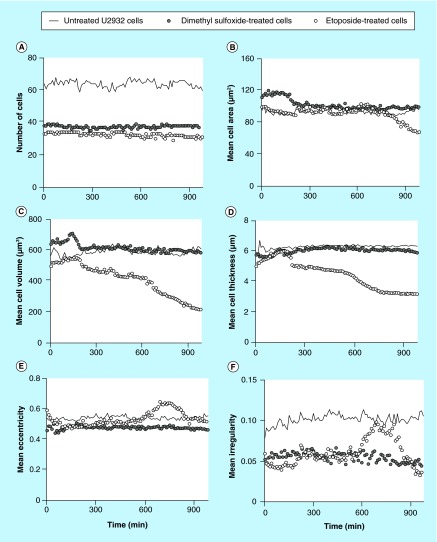
**Different cellular parameters of treated (etoposide or dimethyl sulfoxide) or untreated U2932 cells captured on antibody HLA-DR were analyzed over time.** **(A)** Number of cells; **(B)** mean cell area; **(C)** mean cell volume; **(D)** mean cell thickness; **(E)** mean cell eccentricity; and **(F)** mean cell irregularity.

The novel technique for noninvasive cell analysis used in this study is label-free and enables qualitative and quantitative measurements of cellular shape and optical thickness [1–10]. Digital holographic microscopy (DHM) have previously been evaluated by us, as a cell counting tool for adherent cells and compared with manual counting with a hemocytometer, showing that the technique was comparable with manual counting, with the additional benefits of being automatic, label-free and not damaging to the cells [3]. The recent development in digital holography has been possible due to technical advances in digital sensors and computers [11]. The holographic principle is based on the phenomenon of interference between wave fronts of coherent light scattered by the object studied and an unaffected (not scattered) reference wave front. In digital holography, a digital sensor (e.g., a CCD-sensor) is used for recording and the reconstruction is performed numerically by computer software [12]. In one recording, three images are taken in three different exposures; the object image, the reference image and the hologram image, which is the interference pattern of the object and the reference light [13]. All information needed for 3D reconstruction is contained in the hologram since the focus depth can be altered to any distance after the image has been taken. By reconstructing the image of the object in multiple adjacent planes the 3D image can hence be built up [12].

The balance between cell growth and controlled cell death is very crucial for many physiological processes [14]. Morphologically, dying cells differ from viable cells in many ways, including cell volume changes. The characteristics of apoptosis are a variety of morphological changes such as loss of cell membrane asymmetry and attachment, cell shrinkage, formation of small blebs, nuclear fragmentation, chromatin condensation and chromosomal DNA fragmentation and finally breakdown of the cell into several apoptotic bodies [15,16]. The most commonly used assays to differentiate between viable and non-viable cells today are trypan blue and propidium iodide staining, both laborious and time-consuming methods. Therefore, DHM is now one of the popular technologies that are used by several groups for cancer cell morphology analyses. DHM has recently been used to measure cell volume changes induced by apoptosis with high time and volume resolution, and in real-time [17]. In another study, excessive stimulation of neurotransmitters through addition of l-glutamate was used to induce cell death in primary cultures of mouse cortical neurons [10]. Cell volume regulation was monitored by DHM phase response which allowed estimation in a very short time-frame, if a neuronal cell would survive or die.

To enable DHM analysis of death-induced suspension cells, we have uniquely added antibody-based microarrays [18] to the experimental set-up. High-performing recombinant antibody microarrays have been developed for immunophenotyping, paving the way for large-scale analysis [19]. Indeed, antibody-based microarray techniques have been used to determine discriminating surface antigen (CD) expression profiles for different B-cell populations and their correlations to discrete leukemia subtypes as well as drug target identification of leukemia cells [20–23]. Moreover, Stybayeva *et al*. recently showed that lensfree holographic imaging combined with antibody microarrays can be used for both characterization of T lymphocytes (CD4 vs CD8) and quantification of cytokine signals [24].

The recombinant antibody-based microarrays were used in this study for specific antigen-capture and immobilization of two selected suspension cell lines, a diffuse large B-cell lymphoma (DLBCL) cell line (U2932) and the T-cell acute lymphoblastic leukemia cell line Jurkat, in combination with subsequent analysis with DHM. Results showed a stable cell number over time for both untreated and treated cells. The cell volume was not changed in untreated cells when analyzed for up to 960 min (16 h), whereas for Jurkat T-cells, the etoposide and dimethyl sulfoxide (DMSO) concentrations used in the study resulted in a decline in cell volume after treatment. For the U2932 B-cells, the etoposide-treatment showed a decrease in cell volume after the same time. The results suggest that DHM is advantageous as a future evaluation tool for suspension cell treatments based on the morphological volume changes accompanying cell death.

## Materials & methods

### Cell lines

The Jurkat cell line was from ATCC-LGC (LGC Standards, Teddington, Middlesex, UK), whereas U2932 was obtained from Department of Oncology, Genetics and Pathology, Uppsala, Sweden. Both cell lines were maintained in RPMI 1640 (Invitrogen, San Diego, CA, USA) supplemented with 10% fetal calf serum and 50 µg/ml gentamycin (Invitrogen). The cells were cultured in a humidified atmosphere at 37°C with 5% CO_2_. A volume of cell suspension containing 2 million cells was centrifuged and thereafter re-suspended in 1 ml phosphate-buffered saline (PBS) with 0.5% (w/v) bovine serum albumin (BSA). The cell suspension containing 200,000 cells/100 µl in PBS with 0.5% (w/v) BSA was applied to an antibody array and incubated at room temperature for 30 min. The array was thereafter washed manually less than equal to ten-times with 100 µl PBS-0.5% BSA each time, until no cells were found outside the antibody functionalized spots, in other words the cell binding areas were clearly visible.

### Cell treatment

For each experiment, time-lapse series were run for 960 min (16 h) and holographic imaging was performed every tenth minute. Each experiment was performed at least twice. The experiments were conducted in air without supplemented CO_2_ and at room temperature. To avoid drying, a cover glass was attached over the cells. The cell suspension containing 200,000 cells in 100 µl PBS-0.5% BSA was applied to an antibody array and incubated at room temperature for 30 min. After washing, the antibody coated spots 10 which any cells had specifically bound were clearly and distinctly visible. For untreated cells, 30 µl PBS-0.5% BSA was added to the adhered cells. For treated cells, either 30 µl of 10% DMSO, or 30 µl of 500 µM etoposide (Sigma-Aldrich Co., MO, USA) in PBS-0.5% BSA was added to the array before covering.

### Antibody microarrays

Recombinant antibody arrays containing 13 different single-chain variable antibody fragments (scFv) [25] against five different cell surface membrane proteins and two carbohydrates, plus a negative control (PBS) were immobilized to silane-coated glass slides (Sigma-Aldrich) through passive adsorption. The scFv antibodies were selected from a large phage display library. This library represents a renewable antibody source, and the antibodies have been designed for microarray applications by molecular design, thus displaying high-on chip performance (e.g., stability, reproducibility, functionality and specificity). The probe source (format) has been found to display superior on-chip performances compared with conventional monoclonal and polyclonal antibodies [25,26]. The specific 13 antibodies used in this study were selected based on the criteria that they were tested to work well in microarray applications and directed against target antigens located in the cell surface membrane [19,27,28]. More specifically, we use recombinant scFv antibodies that have been designed for microarray applications by molecular design, thus displaying high-on chip performance. The scFv antibodies can target both high- and low-abundant (pM to fM range) analytes in crude nonfractionated proteomes in a highly specific and reproducible manner (CV < 10%). The scFv antibodies were produced in *Escherichia coli* and purified using Ni^2+^-NTA affinity chromatography as described elsewhere [29].

The purified antibodies were stored in PBS at 4°C until use. The arrays were produced by dispensing 300 pl antibody solution (0.24 ± 0.35 mg/ml) in discrete positions using the noncontact inkjet printer Sci Flexarrayer S11 (Scienion AG, Berlin, Germany). In this study, we printed four subarrays per slide, and each subarray was composed of 14 × 8 individual spots, meaning that 13 antibodies + 1 control was spotted in eight replicates.

### Microscope & software

For cell imaging the HoloMonitor™ M2 (Phase Holographic Imaging AB, Lund, Sweden) was used, which combines both phase contrast microscopy and digital holography. It uses a 0.8 mW HeNe laser (633 nm) with an intensity of approximately 10 Wm^-2^. The exposure time during imaging was less than 3 ms which assures insensitivity to vibrations and minimal physiological effects on cell function. The image algorithm HoloStudio (Phase Holographic Imaging AB) was used to analyze different cell parameters, for example, cell area, cell thickness and cell volume, as described elsewhere [3,6–8].

## Results

### Antibody binding of Jurkat & U2932 cells

The degree of cell binding to the arrayed antibodies was first studied using phase contrast microscopy (Table 1). One cell binding antibody area was selected for holographic photography. The antibody area was selected based on representative cell binding and number of cells bound, over several trials. The number of cells that bound to each antibody spot varied between about 25 and 65, but most spots contained 30 to 40 specifically captured cells. The last criterion was included to avoid two cells being segmented as one because of too close binding. The consistency in the binding patterns could hence be noticed. The Jurkat cells bound to the Lewis X Clone-1 and Clone-2 antibodies and sometimes a weak binding to sialyl Lewis X antibodies could be observed. For Jurkat cells Lewis X Clone-1 antibody was used for holographic measurements. U2932 cells bound consistently to Lewis X Clone-1 and HLA-DR antibodies and in some cases also to CD40 and Lewis Y antibodies. When imaging U2932 cells, HLA-DR or Lewis Y antibody spots were selected.

### Image acquisition & analysis of cell properties

For each time point of holographic measurements, three images were obtained: the object wave image, the reference wave image and the hologram image, which is the interference pattern of the former two, as shown for untreated Jurkat cells (Figure 1A–C). A ‘height map’ (Figure 1D), was performed by the computer software, which subsequently used a segmentation algorithm to find the individual cells enabling analysis of cell parameters (Figure 1E). The segmentation process most often succeeded well in dividing between adjacent cells, but for some samples the focus had to be reset manually to make the image sharp enough for segmentation or the segmentation parameters (e.g., threshold for core thickness) had to be adjusted. Numerical reconstruction of holograms into a 3D image (Figure 1F) was performed by the computer software which subsequently used a segmentation algorithm to find the individual cells enabling analysis of cell parameters.

### Analysis of cell holograms

To investigate the cellular responsiveness, Jurkat and U2932 cells were treated with etoposide, DMSO or left untreated and a cover glass was added to avoid evaporation and keep concentrations constant. Holograms were collected every tenth minute for a period of 16 h. To show images of the holograms after segmentation, four different time points were chosen for Jurkat cells and U2932 cells, respectively. Jurkat cells captured on antibody Lewis X Clone-1 are displayed as control, DMSO and etoposide-treated cells at the time-points 10, 310, 630 and 950 min (Figure 2). An enlargement of the cell area is seen in Jurkat cells after DMSO-treatment. U2932 cells captured on antibody HLA-DR are displayed as control, DMSO and etoposide-treated cells at the timepoints 10, 310, 630 and 950 min (Figure 3).

### Analysis of cell parameters

Data for cell number, cell area, cell thickness, cell volume, cell eccentricity and cell irregularity were collected and the results for the different treatments of Jurkat and U2932 were plotted versus time. Untreated Jurkat cells showed stable cell number over the time period (Figure 4A). The mean cell area for untreated Jurkat cells was also stable and was measured to 110–120 µm^2^ (Figure 4B). When treating the Jurkat cells with either the solvent DMSO or high concentrations of etoposide (500 µM), the mean cell area became unstable. The DMSO treatment initially resulted in an increase of the cell area (also seen in Figure 2), and with time a decrease down to 60–70 µm^2^, which was similar to the final mean cell area of etoposide-treated Jurkat cells. The mean cell volume was initially around 600 µm^3^. The volume of DMSO-treated cells decreased much faster than etoposide-treated cells, but after about 800 min, the cells have reached about the same decrease in mean cell volume. The mean eccentricity of the cells does not show any major variations (Figure 4E), whereas an increase in the mean cellular irregularity was more pronounced for DMSO-treated cells (Figure 4F).

Interestingly, the U2932 cells showed a somewhat different pattern compared with the Jurkat cells. First, the untreated U2932 cells showed stable cell number over the time period (Figure 5A), but the cell number for the treated U2932 cells were lower from the beginning of the experiment (also seen in Figure 3). The mean cell area for the U2932 cells was slightly smaller compared with the area of the Jurkat cells (Figure 5B). The DMSO-treatment resulted in a fast increase of the cell area, but at a later time point the area decreased. The etoposide-treatment resulted in a later increase of the cell area (600 min), which finally declined. The mean cell thickness and volume of treated U2932 cells showed initially a slight increase (Figure 5C). Over the time period of measurement, only the etoposide-treated U2932 cells showed a change in cell volume from 600 µm^3^ down to 200 µm^3^. The mean eccentricity of the U2932 cells does not show any major variations (Figure 5E), whereas etoposide-treated cells showed a peak in increased mean cellular irregularity between 600 and 900 min. (Figure 5F).

Taken together, we showed for the first time that DHM in combination with antibody microarrays could be used to analyze treated nonadherent DLBCL and T-cell acute lymphoblastic leukemia cells in real time.

## Discussion

Development of fast and accurate evaluation tools for cancer treatments will be of great value to clinicians in deciding the most appropriate treatment for patients. The novel technology of DHM uniquely combined with recombinant antibody microarrays presented here provides a whole new ability of specific capture of suspension cells and determination of qualitative and quantitative cellular parameters, combined with a subsequent in real time analysis of induced morphological changes after treatment with cell-death inducing agents. In this study, we have used DHM for determining the cell area, thickness and volume to evaluate the cell death progression in suspension cell lines treated with either etoposide or the solvent DMSO. Experiments were conducted over night with a cover glass attached on top of the antibody microarray to minimize evaporation. Untreated controls for both U2932 and Jurkat cells showed very good stability for measurements up to 16 h, with no changes in the measured cell number. Furthermore, no notable detachment of cells from the antibodies could be detected. The adhesion profile is in agreement with a previous study using the DHM, where U2932 cells were found to adhere strongly to antibodies binding to HLA-DR and Lewis X antibodies (unpublished results). For control cells, the mean cell area, thickness and volume measurements were stable.

Both cell lines were treated with high concentrations of etoposide for induction of fast morphological changes. Changes in cell volume are associated with both normal cellular processes such as proliferation and cell cycle regulation, but also associated with programed cell death [17]. Here, we show that the cell volume of etoposide-treated Jurkat cells and U2932 cells decreases 2–3 h after initiating the treatment. For U2932 cells, the decrease in cell thickness after etoposide-treatment was prominent and also affects the overall cell volume decrease. Etoposide causes errors in the DNA synthesis and promotes apoptosis of the cancer cell by forming a ternary complex with DNA and the enzyme topoisomerase II [30]. Interestingly, the solvent DMSO also resulted in prominent swelling of the Jurkat cells, quite in contrast to the outcome of the U2932 cells.

We also show novel results on the morphological cell parameters eccentricity and irregularity. Interestingly, the Jurkat cells showed a high degree of irregularity after DMSO-treatment. In contrast, the etoposide-treated U2932 cells showed a peak in both cellular eccentricity (unconventional or irregular behavior) and cellular irregularity after 600 min of treatment, indicating the possibility to measure morphological changes at any time point during DHM analysis.

The interest for analyzing cell volume changes of adherent cells with DHM as a result of cytotoxicity or apoptosis treatment has recently increased in popularity [10,17,31–33]. Hematological neoplasms, such as leukemia and lymphoma, are examples of diseases that could benefit from development of the DHM technique. There are many different subtypes of hematological malignancies and several different techniques are used to classify samples from patients, for example, morphology, pathological studies, immunophenotyping, cytogenetics and molecular genetics [34,35]. Although significant advances in diagnostics have been made during the last decades, there are still difficulties with stratification of important disease categories such as DLBCL and follicular lymphoma.

In conclusion, we have provided evidence that suspension cells specifically captured by recombinant antibody-based microarrays, either individual cells or cell populations, could be monitored in real time directly on glass slides, and morphological parameters such as cell area, thickness, volume and irregularity could readily monitored and analyzed.

## Conclusion & future perspective

Different subclasses of leukemia and lymphoma need different treatment protocols involving chemotherapy, radiation therapy and bone marrow stem cell transplantation and the prognosis for different diagnoses is also varying. To improve the outcome for patients with hematological malignancies, new therapies are required, but as a correct diagnosis is crucial for the choice of treatment and hence the outcome. There is also an urgent need for fast and more accurate diagnostic tools. Considerable progress has been made in the DHM field in the past few years, and the use of DHM combined with antibody microarrays as a fast, automatic and cost efficient evaluation tool for different cancer treatments looks promising.

**Table 1.** **Schematic of the array layout and binding of the 13 different single-chain variable antibody fragment fragments directed against two carbohydrates and five different cell surface membrane proteins..**

**Array row**	**Specificity**	**scFv clone**	**scFv concentration (mg/ml)**	**Jurkat binding**	**U2932 binding**
1	CD40 ligand	Clone-1	0.20–0.23	-	-
2	LeX	Clone-1	0.20–0.28	++	++
3	LeX	Clone-2	0.20–0.28	++	-
4	LeY	Clone-1	0.20–0.21	-	+
5	Sialyl LeX	Clone-1	0.20–0.24	+	-
6	CD40	Clone-1	0.28–0.40	-	+
7	CD40	Clone-2	0.20–0.26	-	+
8	CD40	Clone-3	0.20–0.24	-	+
9	HLA-DR	Clone-1	0.20–0.24	-	++
10	ICAM-1	Clone-1	0.20–0.26	-	-
11	IgM	Clone-1	0.20–0.28	-	-
12	IgM	Clone-2	0.20–0.28	-	-
13	IgM	Clone-3	0.20–0.26	-	-
14	Phosphate-buffered saline	-	-	-	-

**Executive summary**

Suspension cell lines can be captured to antibody-based microarrays and thereafter measured for cell number, mean cell area, thickness and volume using digital holographic microscopy.
The cell number was stable over time and the two cell lines showed cell-specific results of cell area and cell irregularity after treatment.
The cell volume could be analyzed in cells treated for up to 16 h, showing a decrease in both cell lines, whereas untreated cells showed stable volume.
Our results provide support for using the concept of digital holography combined with antibody-based microarray technology as a novel method for detecting morphological changes in specifically captured death-induced cells.
